# Structures and host-adhesion mechanisms of lactococcal siphophages

**DOI:** 10.3389/fmicb.2014.00003

**Published:** 2014-01-16

**Authors:** Silvia Spinelli, David Veesler, Cecilia Bebeacua, Christian Cambillau

**Affiliations:** ^1^Architecture et Fonction des Macromolécules Biologiques, UMR 7257, Aix-Marseille UniversitéMarseille, France; ^2^Architecture et Fonction des Macromolécules Biologiques, UMR 7257, Centre National de la Recherche ScientifiqueMarseille, France

**Keywords:** bacteriophage, *Lactococcus lactis*, Siphoviridae, electron microscopy, crystal structure

## Abstract

The Siphoviridae family of bacteriophages is the largest viral family on earth and comprises members infecting both bacteria and archaea. Lactococcal siphophages infect the Gram-positive bacterium *Lactococcus lactis*, which is widely used for industrial milk fermentation processes (e.g., cheese production). As a result, lactococcal phages have become one of the most thoroughly characterized class of phages from a genomic standpoint. They exhibit amazing and intriguing characteristics. First, each phage has a strict specificity toward a unique or a handful of *L. lactis* host strains. Second, most lactococcal phages possess a large organelle at their tail tip (termed the baseplate), bearing the receptor binding proteins (RBPs) and mediating host adsorption. The recent accumulation of structural and functional data revealed the modular structure of their building blocks, their different mechanisms of activation and the fine specificity of their RBPs. These results also illustrate similarities and differences between lactococcal Siphoviridae and Gram-negative infecting Myoviridae.

## INTRODUCTION

*Lactococcus lactis* is a Gram-positive bacterium extensively used for the production of fermented milk products, such as cheese production buttermilk and sour cream. *L. lactis*-containing starter cultures are therefore used world-wide in large scale industrial processes, which create ideal ecological niches for bacteriophage infections and development. These infections have a major economic impact due to impairment of lactococcal fermentations and the resulting need to close and decontaminate the production plants. Addressing this problem has proven to be challenging as *L. lactis* virulent phages are ubiquitous in plant environments and pasteurized milk (Moineau et al., 2002).

Hundreds of *L. lactis* phages strains have been isolated to date and they have been classified into 10 groups (Deveau et al., 2006). The lactococcal phage population is largely dominated by the Siphoviridae family, i.e., phages with a long non-contractile tail, with groups 936, P335 and c2 accounting for ~80, 10, and 5% of these virions, respectively. Only two lactococcal phages from a different family have been isolated belonging to the short-tailed Podoviridae. No Myoviridae infecting *L. lactis* has been reported.

Phages from the c2 group have been shown to recognize and infect *L. lactis* using a protein receptor, the phage infection protein (PIP; Babu et al., 1995; Mooney et al., 2006). PIP is orthologous to the *Bacillus subtilis* protein YueB, a component of type VII secretion system (Abdallah et al., 2007) and the receptor of phage SPP1 (Sao-Jose et al., 2006; Vinga et al., 2012). In contrast, no proteinaceous receptor could be identified for other lactococcal phages, suggesting early on that they may use saccharidic receptors for infection (Valyasevi et al., 1990; Ruud et al., 1994; Deveau et al., 2002). A striking property of lactococcal phages is their narrow host specificity: each of the hundreds of lactococcal phages recognizes only one or a handful of *L. lactis* strains. This observation along with the absence of identified protein receptors supported the hypothesis that non-c2 phages use saccharidic receptors, since only polysaccharides could provide a sufficient diversity to rationalize this data.

This review focuses on the structure of lactococcal phages p2, a lytic phage, and TP901-1, a lysogenic phage, belonging to the predominant 936 and P335 groups, respectively. Their complete structures have been tackled using electron microscopy (EM), and the structure of their components involved in adhesion was determined by X-ray crystallography. These structural data, together with functional studies, made it possible to reveal striking features of lactococcal phages concerning their baseplate activation, and the specificity of their receptor binding proteins (RBPs). Since the recent discovery of the *L. lactis* phospho-polysaccharide “pellicle,” understanding lactococcal phages specificity at molecular levels begins to unravel (Andre et al., 2010; Chapot-Chartier et al., 2010).

## OVERALL PHAGE STRUCTURE

**FIGURE 1:**
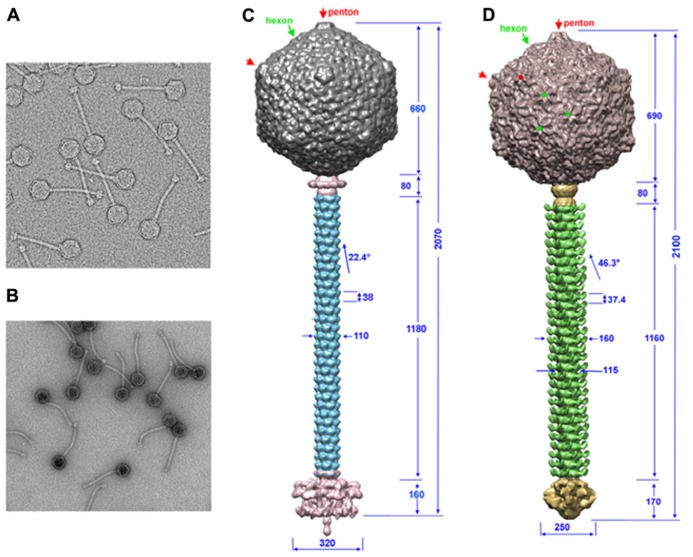
**The TP901-1 and p2 phages assembled structures. (A,B)** Electron microscopy images of phages TP901-1 **(A)** and p2 **(B)**. **(C,D)** The structures of phage TP901-1 **(C)** and p2 **(D)** were generated by assembling the reconstructions of the capsid (top), connector and tail (middle), and the tail-tip (bottom) on low-resolution maps of the full phages. In the capsid map, pentons are identified by red arrows/points and hexons by green arrows/points. Dimensions are given in Å and the angle of rotation between MTP hexamers is given in degrees.

Knowledge on phage structures has to date primarily relied on the structures of Myoviridae or Podoviridae, since the flexible tail of Siphoviridae has prevented the application of single-particle reconstruction in a straightforward manner (**Figures 1A,B**). Lactococcal phages TP901-1 and p2 EM structures could be determined by dissecting the phages in smaller parts: capsid, connector, tail segments, and tail tip. The EM single-particle reconstruction was performed individually and the structures of these parts were reassembled on a scaffold obtained from analysis of a few straight tailed phages (Bebeacua et al., 2013a,b; Sassi et al., 2013). Both phages possess a *T* = 7 *laevo* icosahedral capsid, and the major capsid protein (MCP) hexamer from phage HK97 (Wikoff et al., 2000) fits in the EM map with a satisfying correlation coefficient. No protruding decorations are present on the capsid surface in contrast with what has been reported for some other coliphages, such as T4 (Olson et al., 2001; Fokine et al., 2005). The connector structures of p2 and TP901-1 are similar to that of SPP1, comprising a dodecameric portal (Dube et al., 1993) and the two head-to-tail junction proteins (Lhuillier et al., 2009). The tail structures of phages TP901-1 and p2 are of comparable length, and the major tail protein (MTP) hexamers are of similar thickness, while their helical pitch is significantly different (Bebeacua et al., 2013a,b; **Figures 1C,D**). A striking difference between the two tails is the presence of decorations on the tail of phage p2. Sequence analysis of phage p2 MTP revealed that its N-terminus shares similarity with other MTPs from other phages, such as SPP1 and Lambda (Pell et al., 2009). At the C-terminus it possesses an adhesin fold which appears as decorations on the surface of the tail. In the cases of phages SPP1 (Auzat et al., 2008) and Lambda (Fraser et al., 2006; Pell et al., 2010), it has been suggested that such C-terminal domains help the primary adhesion of phages to their host. Such decorations have also been evidenced in the mycobacterial phage, Araucaria (Sassi et al., 2013). Large baseplate structures are present at the distal tail extremity, which can vary significantly in size and shape, and form the control center for infectivity (Bebeacua et al., 2010; Sciara et al., 2010). In contrast, phages Araucaria (Sassi et al., 2013), SPP1 (Plisson et al., 2007), T5 (Breyton et al., 2013), and Lambda (Davidson et al., 2012; Tam et al., 2013) exhibit a simplified tail tip, in agreement with the fact that these phages have been shown to recognize and attach to a host protein receptor.

## GENOMIC COMPARISONS

**FIGURE 2:**
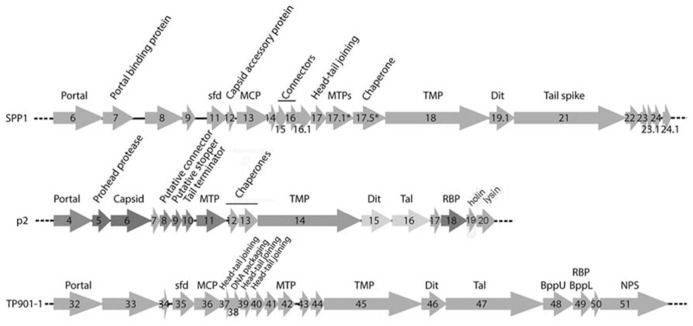
**Schematic representation and assignment of the structural gene module of phages SPP1, p2, and TP901-1.** Genes coding for non-structural ORFs are in light gray. Sfd, scaffolding; MCP, major capsid protein; MTP, major tail protein; TMP, tail tape measure protein; Dit, distal tail protein; Tal, tail-associated lysine; RBP, receptor binding protein; BppU, baseplate upper protein; BppL, baseplate lower protein; NPS, neck passage structure. * indicates alternate splicing.

Phages p2 and TP901-1 have similar structural genomic modules resembling that of phage SPP1 (Chai et al., 1993; Brondsted et al., 2001; Bebeacua et al., 2013b; **Figure 2**). A prominent feature of these modules is the long tape measure protein (TMP), which determines the length of the tail (Pedersen et al., 2000) and participates in the infection mechanism (Boulanger et al., 2008). Upstream, the genes of tail chaperones (Siponen et al., 2009a; Pell et al., 2013), MTP, capsid, and connector proteins are easily identified. Downstream, the first gene encountered is that of the distal tail protein (Dit) conserved in the three phages (Sciara et al., 2010; Veesler et al., 2010, 2012), but also in all Siphoviridae, including those infecting Gram-negative bacteria such as T5 (Flayhan et al., 2014). The X-ray structures of Dit have been determined and are presented below. Downstream the Dit *orf*, *Tal* genes exhibit varying lengths among phages, comprised between ~400 and more than 1000 residues. HHpred (Soding et al., 2005) analysis revealed that the N-terminus (~400 residues) of Tal proteins share a common fold, similar to that of T4 phage gp27 (Kanamaru et al., 2002), and to the type 6 secretion system (T6SS) VgrG module (Leiman et al., 2009). Downstream the Tal *orf*, large differences occur between the genes of the different phages in this region, reflecting the diverging structure of tail tips. In the case of phage p2, it has been demonstrated that the RBP open reading frame (ORF) is the last gene encoding for a structural protein and is located directly upstream the holin and lysin ORFs (**Figure 2**; Ledeboer et al., 2002; De Haard et al., 2005). Considering the sequence similarity between the p2 RBP (ORF18) and the TP901-1 RBP (ORF49), the same can be assumed for the latter phage. These two proteins were subjected to structural and biophysical studies.

While no peptidoglycan-digesting enzyme could be identified within the p2 structural cassette, the C-terminal moiety of the TP901-1 Tal has been shown to possess such an activity. Recent data demonstrated that TP901-1 mutated virions with Tal depleted of the peptidoglycan digesting enzyme domain could still infect their host during the *L. lactis* exponential phase growth, when the cell wall is either not or is less cross-linked to enable rapid cell division. In contrast, TP901-1 native phages possessing the peptidoglycan digesting Tal domain are able to infect the cell, even during the stationary phase (Stockdale et al., 2013).

## RECEPTOR BINDING PROTEIN STRUCTURES

### PHAGE p2

Llama immunization with p2 virions allowed to isolate single-domain llama antibody fragments (named VHH or nanobodies; Hamers-Casterman et al., 1993; Muyldermans et al., 2001) recognizing and neutralizing the p2 RBP. Such a nanobody (VHH5) was located at the distal part of the phage tail using immunogold labeling. Addition of this nanobody to a bacterial culture suppressed phage infection (Ledeboer et al., 2002; De Haard et al., 2005). Furthermore, it was demonstrated that VHH5 was an excellent binder of ORF18 (Kd value of ~1.4 nM), identifying it as the RBP.

**FIGURE 3:**
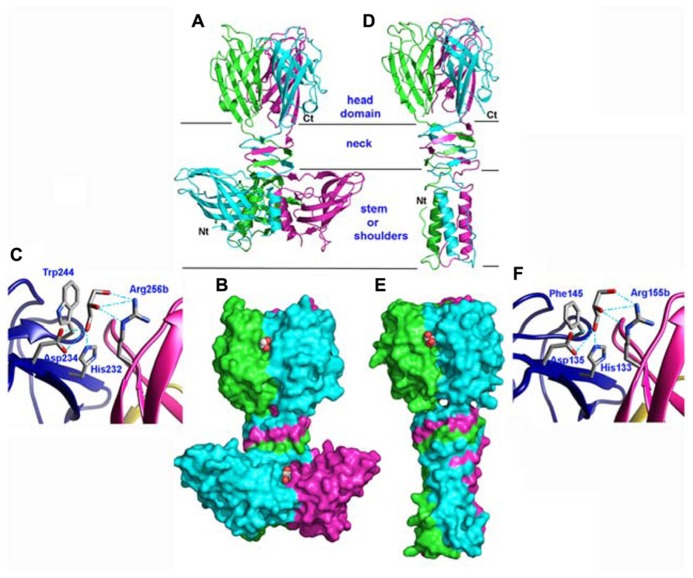
**Structures of the receptor binding proteins (RBPs) of phages p2 and TP901-1. (A,D)** Ribbon view of the p2 RBP trimer **(A)** and of the TP901-1 RBP trimer **(D)**. Monomers are colored green, blue, and pink. **(B,E)** Surface representation of the p2 RBP trimer **(B)** and of the TP901-1 RBP trimer **(E)**. Bound glycerol molecules are represented by spheres (carbon, white; oxygen, red). **(C,F)** Close-up view of glycerol in the receptor binding site of the RBPs of phages p2 **(C)** and TP901-1 **(F)**. The glycerol molecule and the side-chains of the residues participating to binding are represented as sticks (carbon, white; oxygen, red; nitrogen, blue).

With in view to determine the receptor binding site of the p2 RBP, its crystal structure was determined alone and in complex with VHH5 (Spinelli et al., 2006b; Tremblay et al., 2006). The phage p2 RBP is an assembly of three chains of 264 amino acids forming a homotrimer. Noteworthy, a similar trimeric arrangement has also been observed in RBPs of mammalian adenoviruses and reoviruses (van Raaij et al., 1999; Chappell et al., 2002; Burmeister et al., 2004) as well as in the phage T4 gp12 protein (van Raaij et al., 2001). As observed in the phage T4 gp12 trimer (van Raaij et al., 2001), the p2 RBP is organized into three domains: shoulder, interlaced neck and head (**Figures 3A,B**). The shoulder domain (residues 1–141) has a β-sandwich fold assembling two 4-stranded anti-parallel β-sheets. A long helix contributed by each domain allows the three shoulder moieties to associate tightly. Immediately following the shoulder domains, the neck domain forms a triple-stranded β-helix of three segments organized into four β-strands along the threefold symmetry axis (β-prism). This very rigid neck structure has already been observed in the gp12 short tail fiber of *Escherichia coli* phage T4 (Myoviridae family), in which it plays a similar role, linking the N-terminal domain and the receptor binding head (van Raaij et al., 2001). The phage T4 puncturing device also contains a similar structure, but of a much larger diameter (Kanamaru et al., 2002). The RBP head domain follows the neck and forms a β-barrel comprising seven anti-parallel β-strands. Each β-barrel in the trimer is parallel to the threefold axis and interacts with the other two, yielding a very compact structure (**Figures 3A,B**). It exhibits low but significant similarity to RBPs of other viruses: the reovirus attachment protein σ1 trimer (Chappell et al., 2002) and the head domain of the adenovirus fiber (van Raaij et al., 1999).

### PHAGE TP901-1

The phage TP901-1 RBP trimer structure was determined by X-ray diffraction (Spinelli et al., 2006a; Bebeacua et al., 2010). Its N-terminal segment is composed of elongated chains (residues 1–10), β-turns, and a three-helix-bundle assembled through non-polar side chain contacts (**Figures 3D,E**). This “stem” domain is much smaller than the corresponding shoulder domain in the phage p2 RBP (**Figures 3A,B**). However, in the p2 RBP, the three parallel helices (residues 19–32) located close to the threefold axis, are in a similar location to those of the TP901-1 RBP (**Figures 3A,D**).

Following the helix bundle domain, a short linker structure (residues 31–39) connects the α-helical domain (17–30) and the β-prism (40–63; **Figures 3D,E**). The β-prism neck domain interlaces three segments of each subunit, and each of its three faces is made of four β-strands from the three monomers, constituting a domain comparable to that of the RBP of phage p2 (Spinelli et al., 2006b) and to a streptococcal lyase (Smith et al., 2005). The amino acid sequence of the TP901-1 RBP neck region exhibits a six residue long, regular and repeating motif, not observed in phage T4 gp12 nor in the RBP neck of phage p2. Each segment ends with a polar residue, except the last one where it is replaced by a proline, which redirects the peptide chain upward, similar to the p2 RBP (Spinelli et al., 2006b).

The RBP head domain of TP901-1 (residues 64–163; **Figure 3D**) is a β-barrel formed of anti-parallel β-strands. This domain is the only part that shares sequence similarity with the RBP of p2 (28% sequence identity), and they exhibit very similar structures (**Figures 3A,D**). Noteworthy, the crystal structure of the RBP head domain from phage bIL170 (936 group) displays the same fold as in phages p2 and TP901-1 (Ricagno et al., 2006). The modular nature and interchangeability of RBP domains has been demonstrated by producing a chimeric RBP in which the N-terminal and linker RBP domains (stem and neck) of phage TP901-1 were fused to the C-terminal RBP head domain of phage p2 (Siponen et al., 2009b). The structure of this chimera has been determined by X-ray crystallography and it exhibits a stable conformation that closely resembles the parental structures, while a slight displacement of the linker improves the domains junction. Indeed, the receptor-binding site is structurally indistinguishable from that of native p2 RBP and the chimera binds glycerol with equal affinity (see below).

## THE RECEPTOR-BINDING SITE

### PHAGE p2

The high-resolution structure of the p2 RBP (1.7 Å resolution) revealed the presence of three glycerol molecules (originating from the cryoprotectant liquor) bound at the interface between the head domains (Tremblay et al., 2006). The glycerol molecules are tightly bound (B-factors of 17.8 Å^2^) via three hydrogen bonds established between the His 232 and Asp 234 side-chains and the glycerol O1 atom, and between the Arg 256 (from the other monomer) guanidyl group and the glycerol O2 atom (**Figure 3C**). Furthermore, the hydrophobic face of glycerol packs against Trp 244 side-chain, as often observed with sugar complexes (Bourne et al., 1990). Considering the close vicinity of Trp residues to glycerol molecules (Trp 244 and Trp 43), fluorescence quenching experiments made it possible to measure the affinity of the RBP glycerol binding site with glycerol and four different saccharides, and their Kd values ranged from 0.26 to 0.13 μM (Tremblay et al., 2006).

**FIGURE 4:**
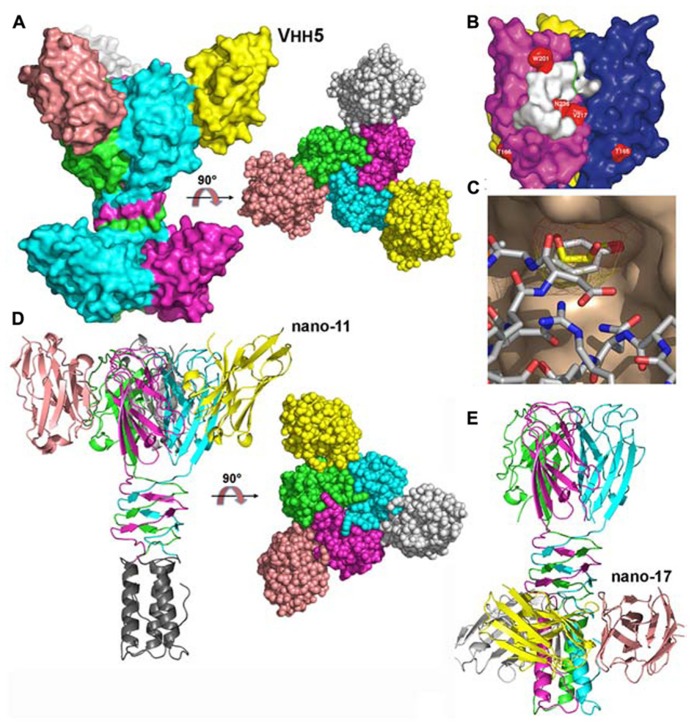
**Structures of the receptor binding proteins (RBPs) of phages p2 and TP901-1 in complex with VHHs/nanobodies. (A)** Surface representation of the p2 RBP trimer in complex with the neutralizing VHH5 (nano5), and 90° rotated view. **(B)** Surface footprint of VHH5 on the RBP trimer surface (white). Mutated residues leading to neutralization escape are indicated in red. **(C)** View of the superposition of the VHH5 Tyr 55 with glycerol. The RBP surface is colored beige, the glycerol carbon atoms are yellow, while those of VHH5 are white. Oxygen atoms are red and nitrogen atoms are blue. **(D)** Ribbon view of the TP901-1 RBP trimer in complex with the neutralizing nanobody 11 and surface view at 90° (right). **(E)** Ribbon view of the TP901-1 RBP trimer in complex with the non-neutralizing nanobody 17. Panels **(B,C)** taken from Tremblay et al. (2006). Copyright © American Society for Microbiology.

The structure of the complex between p2 RBP and VHH5 was also determined by X-ray crystallography and revealed that the nanobody covers a large area of the head domain (Tremblay et al., 2006; **Figures 4A,B**). A specific interaction is observed between Tyr 55 of the nanobody, penetrating deeply in the glycerol-binding site (**Figure 4C**), at the exact position occupied by the latter in the isolated RBP structure, and establishing interactions with Trp 244a and Arg 256b on either side. The OH group from Tyr 55 superimposes with the OH1 of glycerol and establishes similar hydrogen bonds with His 232 and Asp 234.

### PHAGE TP901-1

Glycerol molecules were also observed bound at the interface between head domains in the crystal structure of the phage TP901-1 RBP at 1.6 Å resolution (Spinelli et al., 2006a). Glycerol molecules (**Figure 3F**) are stacked against Phe 145 and establish hydrogen bonds with His 133, Asp 135, and Arg 155. As inferred from sequence alignments, three of these residues in the TP901 RBP are identical to those in the RBP of phage p2, while the fourth one corresponds to a substitution of Phe 145 by Trp 244. Two hydroxyl groups from the glycerol molecule are therefore strongly bound to the RBP head. In contrast, the third hydroxyl group is free and points to the bulk solvent in both structures (**Figure 3F**). This orientation strongly suggests that the saccharidic binding site harbors the terminal residue of the receptor polymer, the free hydroxyl group pointing in the direction of the rest of the receptor polymer attached to the host.

The superposition of the binding network of glycerol in both RBPs shows their striking similarity (**Figures 3C,F**). Differences in amino acid residues are only observed in the second binding sphere, which modulates the binding crevice surface and volume and, hence, may influence the saccharidic specificity observed between different phages and bacterial strains. The absence of tryptophan residue in the vicinity of the glycerol-binding site in the phage TP901-1 RBP prevented us to directly perform binding studies using tryptophan fluorescence quenching. A Phe145Trp point mutant was therefore designed and characterized the affinity of the complexes formed with glycerol, phospho-glycerol, *N*-acetyl muramic acid, muramyl-dipeptide, and galactose. The obtained affinity constants were roughly comparable to those measured for the p2 RBP (Spinelli et al., 2006a).

The structures of the TP901-1 RBP in complex with nanobodies (Desmyter et al., 2013) and designed ankyrin repeat proteins (DARPins; Veesler et al., 2009) were also obtained. In the first case, the, TP901-1 baseplate (see below) was used for llama immunization. Among the different binders characterized, three of them targeted the RBP: nanobodies 2, 11, and 17. Functional studies demonstrated that nanobodies 2 and 11 could neutralize phage infection, while nanobody 17 could not (Desmyter et al., 2013). Indeed, the binding sites of nanobodies 2 and 11 were localized at the interface between head domains, occupying the glycerol binding site (**Figure 4D**). In contrast, nanobody 17 binds the stem domain and does not interfere with the receptor binding site (**Figure 4E**; Desmyter et al., 2013). In the second case, DARPin binders were generated against a subcomplex of the TP901-1 baseplate (i.e., the BppU/RBP tripod complex). We isolated three binders and demonstrated they targeted the RBP and in turn neutralized phage infection (Veesler et al., 2009). The structure of one of them in complex with RBP was obtained revealing a totally different binding mode compared to the VHH/nanobody complexes. A unique DARPin binds at the tip of the RBP head domain, leaving the receptor binding site free, but probably blocking the direct interaction with the host (see below the baseplate section; Veesler et al., 2009).

## THE SACCHARIDIC RECEPTORS AND THEIR INTERACTIONS WITH THE RBPs

**FIGURE 5:**
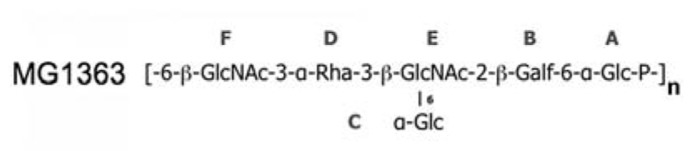
**The “pellicle” phospho-polysaccharide from *L. lactis* MG1363 ()**. This phospho-polysaccharide is the receptor of lactococcal phages sk1 and p2.

The affinity of glycerol and phospho-glycerol for the RBPs suggested that lipoteichoic acids (LTA) could act as receptors for lactococcal phages. Conversely, the observation that many sugars bind equally well to the RBPs and the fact that the structure of LTAs is too simple to explain the different specificities of hundreds of lactoccocal phages constituted arguments against this hypothesis. A recent report by Chapot-Chartier et al. (2010) revealed that the surface of the *L. lactis* cell wall is covered by a “pellicle,” constituted of hexasaccharide phosphate repeating units that are distinct from any other bacterial polysaccharides, which appears as a strong candidate to allow phage adsorption (**Figure 5**). Indeed, *L. lactis* mutants lacking this “pellicle” could not be infected by their specific phage and genomic studies demonstrated the presence of *L. lactis* strain-specific “pellicle” cassettes coding for glycosyl-transferases and other enzymes involved in polysaccharide phosphate synthesis (Mahony et al., 2013). Finally, the diversity induced by ~6-mer saccharides is fully compatible with the fine specificity of lactococcal phages.

Preliminary experiments using surface plasmon resonance (SPR) explained the specificity mechanism of phage p2 RBP for *L. lactis* strain MG1363 (Bebeacua et al., 2013b). The purified pellicle of this *L. lactis* strain was biotinylated and attached to a SPR chip (ligand) while either the RBP of phage p2 or of phage TP901-1 was injected (analyte) to monitor interactions. While a typical saturation curve was obtained for the p2 RBP, yielding a Kd value of 230 ± 40 nM, it was not possible to reach saturation with the TP901-1 RBP. More significantly, the dissociation time of the TP901-1 RBP with the *L. lactis* MG1363 pellicle is extremely short as compared to that of the p2 RBP. As a result, phage TP901-1 would remain in contact for a very short time with the specific host of phage p2, which would be insufficient for adhesion and further infection (Bebeacua et al., 2013b).

## BASEPLATE STRUCTURES AND MECHANISMS OF ACTIVATION

### THE PHAGE p2 BASEPLATE

**FIGURE 6:**
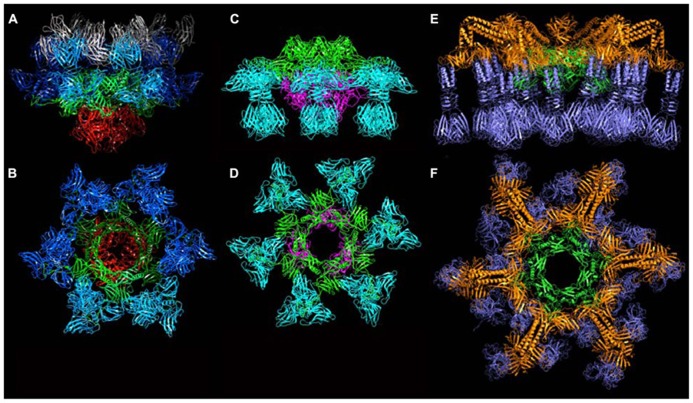
**The crystal structures of the baseplates of phages p2 and TP901-1. (A)** Side-view of the phage p2 baseplate rest form in complex with the llama VHH5 (ORF15/Dit, green; ORF16/Tal, red; ORF18/RBP, blue; VHH5, gray). **(B)** Top-view of the phage p2 baseplate rest form (same colors are in **A**, but the VHH5 has been removed from the view). **(C)** Side-view of the phage p2 baseplate Sr^2^^+^/Ca^2^^+^ activated form (same colors as in **A**). **(D)** Top-view of the phage p2 baseplate Sr^2^^+^/Ca^2^^+^ activated form. **(E)** Side-view of the phage TP901-1 baseplate (ORF46/Dit, green; ORF48/BppU, orange; ORF49/RBP, violet). **(F)** Side-view of the phage TP901-1 baseplate (same colors as in **E**).

Based on their genomic location, we hypothesized that *orfs* 15 (Dit), 16 (Tal), 17, and 18 (RBP) encoded baseplate-related proteins (**Table 1**). The contiguous cluster of four genes was cloned, expressed in *E. coli* and purified, yielding a macromolecular complex of ~1.0 MDa containing ORFs 15, 16, and 18 (Campanacci et al., 2010). ORF17 could not be detected in this assembly, in agreement with its absence in mature virions. Although crystals of the complex were obtained readily, they did not diffract beyond 8 Å resolution. In contrast, after mixing with an excess of VHH5, a new complex was obtained that crystallized and diffracted to 2.6 Å resolution (Sciara et al., 2010). The baseplate-VHH5 (BP-VHH) structure is 230 Å wide and 160 Å high, displays a quasi hexagonal symmetry, and is formed from bottom to top by three ORF16, six ORF15, and six trimers of ORF18, as well as 18 VHH5 (**Figures 6A,B**).

**Table 1:** List of the components of the baseplates from lactococcal phages mentioned in this review.

Protein\phage	Abbreviations	p2	TP901-1	Tuc2009
Tape measure protein	TMP	ORF14	ORF45	ORF48
Distal tail protein	Dit	ORF15	ORF46	ORF49
Tail associated lysozyme	Tal	ORF16	ORF47	ORF50
Baseplate protein (upper)	BppU	n/a	ORF48	ORF51
Baseplate protein A	BppA	n/a	n/a	ORF52
Receptor binding protein Baseplate protein (lower)	RBP (BppL)	ORF18	ORF49	ORF53

**FIGURE 7:**
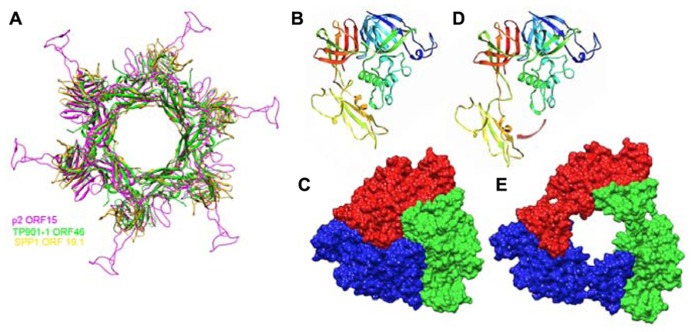
**Crystal structures of components of phages baseplates. (A)** Superimposition of the Dit hexamers structures from phages TP901-1 (ORF46, green), p2 (ORF15, purple), and SPP1 (gp19.1, gold). **(B)** Ribbon view of the crystal structure of ORF16/Tal from the p2 baseplate in the rest form (rainbow coloring, from blue to red). **(C)** Surface view of the closed ORF16/Tal trimer from the p2 baseplate in the rest form. **(D)** Crystal structure of ORF16/Tal from the p2 baseplate in the Sr^2^^+^/Ca^2^^+^ activated form. Domain 4 has moved away from the rest of the molecule. **(E)** Surface view of the open ORF16/Tal trimer from the p2 baseplate in the Sr^2^^+^/Ca^2^^+^ activated form.

ORF15 (Dit) is composed of two domains. The N-terminal domain (“ring domain” 1–132) shows a split barrel-like fold similar to that found in phage Lambda gpV (Pell et al., 2009) and Hcp, a T6SS protein (Jobichen et al., 2010; Veesler and Cambillau, 2011). A long kinked extension (the “belt”) of four β-strands embraces the next ORF15 molecule in the hexameric ring (**Figure 7A**). The N-terminal domains form a tight ring with two layers of β-strands. This ring delineates a 40 Å-wide channel to allow the transit of the dsDNA genome during infection. The C-terminal domains (residues 137–275) are located at the ring periphery, and do not contact each other (**Figure 7A**). They display a galectin fold supplemented by a long extension (the “arm,” residues 147–188) having a critical role in baseplate assembly via the formation of a three-digit hand that anchors the N-terminal domain of the RBP (ORF18, see below; **Figure 7A**).

ORF16 (Tal) is a 398 residue-long protein harboring four domains (**Figures 7B,C**) and its fold is similar to gp27 of myophage T4 (Kanamaru et al., 2002). In contrast to gp27, the ORF16 trimer forms a dome at the distal extremity of the baseplate, thereby closing its central channel (**Figures 6B** and **7C**).

The structure of ORF18 (RBP) is similar to that of ORF18 crystallized alone, with one exception: the N-terminal residues 2–17 of ORF18 in the baseplate structure are ordered and visible in the electron density. This is due to a tight interaction with the three-digit hand from the ORF15 galectin domain (**Figure 7A**). Furthermore, residues 2–7 of ORF18 protrude from each subunit, forming the first strand of the shoulder domain of the next subunit by domain-swapping (Sciara et al., 2010). As with the isolated protein, each ORF18 trimer is coordinated by three VHH5. The 18 VHH5 molecules together with the head domains of ORF18 build a large complex assembled through tight protein–protein contacts that stabilize the ORF18 position and that likely led to better diffracting crystals. Each ORF16 contacts two ORF15, which in turn attach two ORF18 trimers. There are no contacts between ORF16 and ORF18, and therefore, ORF15 hexamer plays the role of a central hub to which ORF16 and ORF18 are attached (**Figures 6A,B**).

The structure of the baseplate reported above exhibited an unexpected conformation. Indeed, one would have expected the head domains of the RBPs (ORF18), which harbor the receptor-binding sites, to point “downward,” i.e., in the direction of the host cell surface. Instead, the RBPs were observed in a “heads-up” conformation, a position not compatible with optimal adhesion. However, the baseplate crystal structure fitted without rearrangement in the baseplate region of the p2 virion reconstruction (Sciara et al., 2010).

**FIGURE 8:**
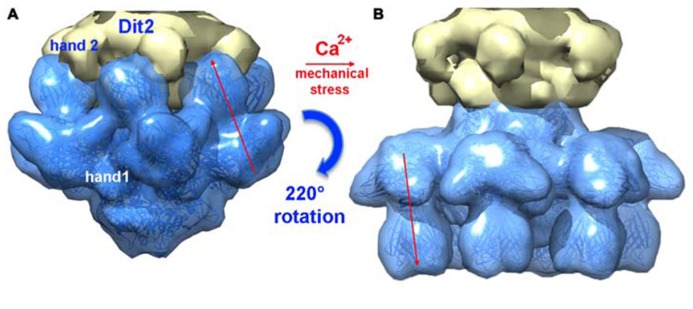
**A composite X-ray/EM reconstruction of the p2 baseplate. (A)** A 20 Å electron density map (blue; ribbon structure inside) of the rest form (free virion) of the p2 baseplate crystal structure was calculated and subtracted from the baseplate experimental EM map. The resulting difference map (yellow) corresponds to a Dit (ORF15) hexamer. **(B)** A 20 Å electron density map (blue; ribbon structure inside) of the activated form of the p2 baseplate crystal structure was calculated and appended to the upper Dit EM map (yellow). Figure adapted from Bebeacua et al. (2013b). Copyright © American Society for Microbiology.

**FIGURE 9:**
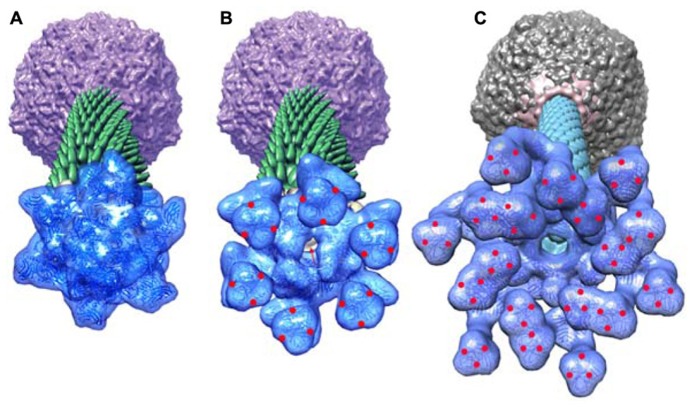
**Perspective views of the reconstructions of the p2 phage. (A)** The baseplate rest form and **(B)** the Ca^+^^+^ activated form, showing in forefront the baseplate structure, closed and opened, respectively. **(C)** The TP901-1 baseplate. The red dots are located at the RBP saccharides binding sites in the activated phage p2 and in the phage TP901-1 representations. Red arrow identifies the open channel of phage p2 activated baseplate. Panels **(A,B)** adapted from Bebeacua et al. (2013b). Copyright © American Society for Microbiology.

Since it was noticed that in some cases lactococcal phages infection required Ca^2^^+^, attempts were made to obtain crystals in the presence of Ca^2^^+^ or Sr^2^^+^ without VHH5. New crystal forms were obtained readily with both cations, and their structure determined. Both structures were found to be identical, although the Sr^2^^+^ complex diffracted to higher resolution (Sciara et al., 2010). The complex comprised six ORF15, three ORF16, and six ORF18 trimers (**Figures 6C,D**). The most striking feature was that the RBPs have rotated by ~200°, to orient their receptor-binding sites toward the distal phage extremity, leading to a “heads down” conformation (**Figures 8** and **9A,B**). The ring formed by the N-terminal domain of ORF15 superposed well between the Sr^2^^+^-free and Sr^2^^+^-bound structures, but the galectin, arm and hand domains had moved significantly (Sciara et al., 2010). The Sr^2^^+^ ion (or Ca^2^^+^ ion) is located at the interface between the N-terminal and the galectin domains of ORF15, and is coordinated by side chains of residues Asn10, Asp12, Asn241, Asp246, and the main-chain carbonyl of Leu11 (Sciara et al., 2010). The arm domains have rotated so that they are oriented in opposite direction compared to the “heads-up” structure. The ORF16 trimer was strongly affected, resulting in the opening of the dome with the concomitant formation of a channel of ~32 Å diameter (**Figures 6D, 7D,E**, and **9A,B**), large enough for dsDNA passage. This opening results from a outward rotation of ORF16 cores with respect to the channel axis, and the opening of a crevice between domains 1, 2, 4, and 3 in an iris-like movement (**Figures 7D,E**). Domain 3 remains in close contact with the next ORF16 in the trimer (**Figure 7E**). In the activated baseplate structure, extensive interactions are established between ORF16s and ORF18s whereas these protein components were not in contact in the BP-VHH structure. In fact, these contacts lock the ORF18s in their “heads-down” conformation, giving to ORF16 the role played by the VHH5 molecules in the BP-VHH complex. Remarkably, the head domains are also maintained by the arm extensions belonging to the proximal Dit domains in the virion before being released when activation occurs (**Figure 8**).

### THE PHAGE TP901-1 BASEPLATE

**FIGURE 10:**
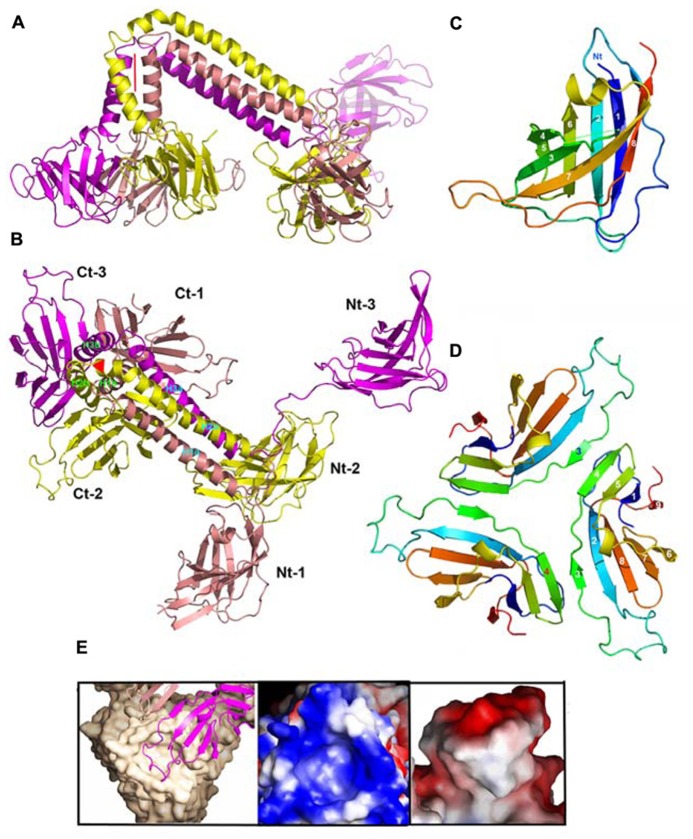
**Structures of components of phage TP901-1 baseplate. (A)** Lateral ribbon view of the ORF48 trimer (salmon, yellow, and violet, for monomers 1, 2, and 3, respectively). **(B)** View 90° from **(A)** (top view) of ORF48 trimer. The N- and C-terminal domains are labeled, 1, 2, 3, respectively. **(C)** The ribbon view (rainbow colored) of the N-terminal domain of ORF48. **(D)** The ribbon view (rainbow colored) of a trimer of the C-terminal domain of ORF48. **(E)** Left to right: Close-up view of the electrostatic surface potential of the interacting regions from BppU and the RBP highlighting their high charge and surface complementarity. Each RBP trimer (beige) is anchored to the baseplate via a loop extending from each BppU C-terminal domain (pink) that penetrates the cup formed at the top of this former protein.

Following the same strategy employed for phage p2, attempts were made to express the phage TP901-1 baseplate by cloning a segment encompassing the *orfs* located between the *dit* and the *rbp* genes (*orfs* 46–49). Although this strategy was unsuccessful, a complex comprising only ORFs 46, 48, and 49 (without Tal, ORF47), could be expressed and purified, and its crystal structure determined at 3.8 Å resolution (Campanacci et al., 2010; Shepherd et al., 2011; Veesler et al., 2012). The TP901-1 baseplate is 320 Å wide and 160 Å high, exhibiting an overall sixfold symmetry, and a mass of 1.76 MDa (**Figures 6E,F**). From the proximal to distal end, it is formed by a Dit hexamer (ORF46) surrounded by 18 copies of BppU (ORF48) holding 54 RBPs (ORF49) organized as 18 trimers (**Figures 10A–D**). All together, it forms a complex of 78 proteins and 54 possible receptor binding sites, organized in six tripods each containing three BppU and three trimeric RBPs (**Figure 9C**; Veesler et al., 2012). Noteworthy, immunization of llamas with this baseplate complex led to tens of nanobody binders, among which only two proved to definitively neutralize phage infection (Desmyter et al., 2013).

The Dit forms a hexameric circular-shaped core with a 80 Å diameter, which delineates a 37 Å wide central channel (**Figures 6E,F**). Six domains are appended to this core without forming contact with each other (**Figure 7A**). Each monomer is formed from a N-terminal domain (residues 1–145) with a β-sandwich, an α-helix, and a β-hairpin, followed by a C-terminal domain (residues 146–255) folded as a galectin-like β-sandwich (Sciara et al., 2010; Veesler et al., 2010; **Figure 7A**). This Dit structure is similar to that of phages SPP1 (Veesler et al., 2010) and p2 (Sciara et al., 2010), demonstrating that this module forms the adsorption apparatus hub in phages of Gram-positive bacteria (Veesler and Cambillau, 2011) and beyond (Flayhan et al., 2014).

The 18 BppU assemble as six asymmetric trimers connecting the Dit central core and the RBPs (**Figures 6E,F** and **10A,B**). Each monomer is composed of a N-terminal globular domain (1–122; **Figure 10C**), a linker (123–134), two helices joined by a kink (135/139–194) and a globular C-terminal domain (195–299; Veesler et al., 2012). The C-terminal domains fold as β-sandwiches and assemble as a threefold symmetric triangular-shaped trimer held via two types of antiparallel pairing (**Figure 10D**). This structure binds to the three stem domains of three RBP trimers (**Figure 6F**). Each BppU C-terminal domain deeply projects a loop (residues 217–234) in the crevice formed at the top of the RBP trimer to anchor it to the baseplate via electrostatic interactions (**Figure 10E**). Moreover, three aliphatic/aromatic residues belonging to BppU (Ile 219, Phe 226, and Phe 232) fill the center of the RBP crevice. The conservation of the residues involved in the BppU/RBP interactions suggests that common architectural themes are found among P335-phages (Veesler et al., 2012).

The RBP structure is identical to the structure of its isolated form, with the three domains forming a trimer (Bebeacua et al., 2010). The three RBPs within each tripod are separated by at least 20 Å whereas extensive inter-tripod contacts involving the 12 most internal RBPs are observed (**Figure 6F**). The six most peripheral RBPs do not establish any contact and appear to be highly mobile (Bebeacua et al., 2010; Veesler et al., 2012).

This baseplate structure is most probably shared, more or less closely, by several phages from the P355 group and beyond. EM studies have demonstrated the structural resemblance of the P335 phage Tuc2009 baseplate with that of TP901-1 (Sciara et al., 2008; Collins et al., 2013). The major difference between these two baseplates is the presence of an extra protein termed BppA in the Tuc2009 baseplate whose gene is located between *bppU* and *bppL*. It was shown that this protein increases the binding specificity and/or affinity of the Tuc2009 tripod to its host receptor (Collins et al., 2013). Although the overall sequence identity between Tuc2009 and TP901-1 phage genomes is higher than 95%, the RBPs differ significantly: the stem and neck domains display high sequence identity, while the head domain displays no identity at all. Indeed, both phages target different *L. lactis* strains (UC509.9 and 3107, respectively), which likely harbor different pellicles in terms of composition and structure.

## ADHESION MECHANISMS OF LACTOCOCCAL PHAGES

**FIGURE 11:**
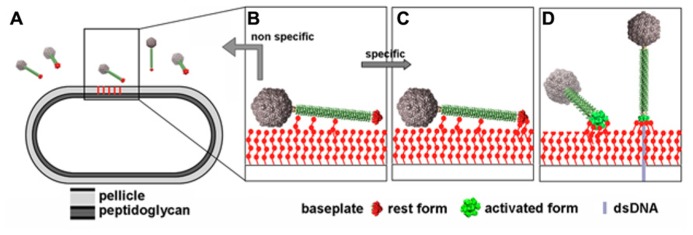
**Putative infection mechanism of *L. lactis* MG1363 by phage p2. (A)** The phages in the vicinity of the host. **(B)** Weak interactions are established between the tail adhesins and putatively the pellicle. **(C)** Strain-specific lateral interactions may occur between the phage RBPs of the resting baseplate and the specific pellicle, leading, in the presence of Ca^+^^+^ to **(D)** baseplate activation, RBPs rotation, and strong binding involving several of the 18 saccharide binding sites. Figure taken from Bebeacua et al. (2013b). Copyright © American Society for Microbiology.

Comparison of the structures of the p2 and TP901-1 baseplates revealed that the latter is already in a “ready to adsorb” conformation without requiring any conformational change. This observation could be correlated with functional data as p2-like phages are non-infectious in the absence of Ca^2^^+^ whereas TP901-1-like phages do not require Ca^2^^+^ for infection (Veesler et al., 2012). These results could be explained by the presence of a conserved Ca^2^^+^-binding loop in the Dit of p2-like phages that is absent in TP901-1-like phages, allowing to rationalize the different activation mechanisms exhibited by these different lactococcal phage families. Furthermore, before specific interactions elicited by the baseplate/pellicle binding occur, a specific binding should maintain the phage long enough in the vicinity of its host in order to scout the cell wall for the proper receptor. In siphophages SPP1 (Auzat et al., 2008) and Lambda (Fraser et al., 2006; Pell et al., 2010) affinity modules have been described in the tail that exert this role. Whereas phage TP901-1 is devoid of these modules, phage p2 possess a tail decorated with such modules (Bebeacua et al., 2013b). This observation leads to suggest a more complete and realistic mechanism for phage p2 adhesion to its host (**Figure 11**).

## CONCLUDING REMARKS AND PERSPECTIVES

The fine specificity of lactococcal phages for their *L. lactis* host strains can now be rationalized considering the diversity allowed by the chemical structure of the “pellicle” phospho-polysaccharide forming the most external layer surrounding these cells. Interactions between phage RBPs and the pellicle appear to be characterized by a moderate affinity but with long enough adhesion times (thanks to low k_off_) to allow initiating infection. Progresses are being made to decipher the RBP/pellicle recognition mechanisms in different phages, aiming to understand the molecular determinants of this specificity.

According to the literature, a large number of phages exhibit a baseplate, beyond those infecting *L. lactis*. This probably reflects the fact that many phages use sugars as receptors. The nominal weaker affinity for polysaccharides as compared to protein/protein interactions (e.g., phage T5 pb5 binds to FhuA receptor with sub-nanomolar affinity; Flayhan et al., 2012) is compensated by a large number of receptor binding sites (18 for p2, 54 for TP901-1), although we do not know if all of them are available for binding (Desmyter et al., 2013). However, due to avidity, binding of only a few receptors should be sufficient to yield a sub-nanomolar Kd. The activation mechanism probably acts as a safety switch allowing promotion of an infection-competent metastable conformation of the virions only when the physicochemical conditions correspond to those of the host ecosystem. The more stable rest state might be well adapted to dissemination of the virions as aerosols. This mechanism, shared by Myoviridae, may encompass a wider range of Siphoviridae beyond lactococcal phages.

## Conflict of Interest Statement

The authors declare that the research was conducted in the absence of any commercial or financial relationships that could be construed as a potential conflict of interest.

## References

[B1] Abdallah A. M. Gey Van Pittius N. C. Champion P. A. Cox J. Luirink J. Vandenbroucke-Grauls C. M. (2007). Type VII secretion – mycobacteria show the way. *Nat. Rev. Microbiol.* 5 883–891 10.1038/nrmicro1773 17922044

[B2] Andre G. Kulakauskas S. Chapot-Chartier M. P. Navet B. Deghorain M. Bernard E. (2010). Imaging the nanoscale organization of peptidoglycan in living *Lactococcus lactis* cells. *Nat. Commun.* 1 1–8 10.1038/ncomms1027 20975688 PMC2964452

[B3] Auzat I. Droge A. Weise F. Lurz R. Tavares P. (2008). Origin and function of the two major tail proteins of bacteriophage SPP1. *Mol. Microbiol.* 70 557–569 10.1111/j.1365-2958.2008.06435.x 18786146

[B4] Babu K. S. Spence W. S. Monteville M. R. Geller B. L. (1995). Characterization of a cloned gene (pip) from *Lactococcus lactis* required for phage infection. *Dev. Biol. Stand.* 85 569–575 8586234

[B5] Bebeacua C. Bron P. Lai L. Vegge C. S. Brondsted L. Spinelli S. (2010). Structure and molecular assignment of lactococcal phage TP901-1 baseplate. *J. Biol. Chem.* 285 39079–39086 10.1074/jbc.M110.175646 20937834 PMC2998104

[B6] Bebeacua C. Lai L. Vegge C. S. Brondsted L. Van Heel M. Veesler D. (2013a). Visualizing a complete Siphoviridae member by single-particle electron microscopy: the structure of lactococcal phage TP901-1. *J. Virol.* 87 1061–1068 10.1128/JVI.02836-12 23135714 PMC3554098

[B7] Bebeacua C. Tremblay D. Farenc C. Chapot-Chartier M. P. Sadovskaya I. Van Heel M. (2013b). Structure, adsorption to host, and infection mechanism of virulent lactococcal phage p2. *J. Virol.* 87 12302–12312 10.1128/JVI.02033-13 24027307 PMC3807928

[B8] Boulanger P. Jacquot P. Plancon L. Chami M. Engel A. Parquet C. (2008). Phage T5 straight tail fiber is a multifunctional protein acting as a tape measure and carrying fusogenic and muralytic activities. *J. Biol. Chem.* 283 13556–13564 10.1074/jbc.M800052200 18348984

[B9] Bourne Y. Roussel A. Frey M. Rouge P. Fontecilla-Camps J. C. Cambillau C. (1990). Three-dimensional structures of complexes of *Lathyrus ochrus* isolectin I with glucose and mannose: fine specificity of the monosaccharide-binding site. *Proteins* 8 365–376 10.1002/prot.340080410 2091026

[B10] Breyton C. Flayhan A. Gabel F. Lethier M. Durand G. Boulanger P. (2013). Assessing the conformational changes of pb5, the receptor-binding protein of phage T5, upon binding to its Escherichia coli receptor FhuA. *J. Biol. Chem.* 288 30763–30772 10.1074/jbc.M113.501536 24014030 PMC3798546

[B11] Brondsted L. Ostergaard S. Pedersen M. Hammer K. Vogensen F. K. (2001). Analysis of the complete DNA sequence of the temperate bacteriophage TP901-1: evolution, structure, and genome organization of lactococcal bacteriophages. *Virology* 283 93–109 10.1006/viro.2001.0871 11312666

[B12] Burmeister W. P. Guilligay D. Cusack S. Wadell G. Arnberg N. (2004). Crystal structure of species D adenovirus fiber knobs and their sialic acid binding sites. *J. Virol.* 78 7727–7736 10.1128/JVI.78.14.7727-7736.2004 15220447 PMC434083

[B13] Campanacci V. Veesler D. Lichiere J. Blangy S. Sciara G. Moineau S. (2010). Solution and electron microscopy characterization of lactococcal phage baseplates expressed in Escherichia coli. *J. Struct. Biol.* 172 75–84 10.1016/j.jsb.2010.02.007 20153432

[B14] Chai S. Szepan U. Luder G. Trautner T. A. Alonso J. C. (1993). Sequence analysis of the left end of the *Bacillus subtilis* bacteriophage SPP1 genome. *Gene* 129 41–49 10.1016/0378-1119(93)90694-X 8335259

[B15] Chapot-Chartier M. P. Vinogradov E. Sadovskaya I. Andre G. Mistou M. Y. Trieu-Cuot P. (2010). Cell surface of *Lactococcus lactis* is covered by a protective polysaccharide pellicle. *J. Biol. Chem.* 285 10464–10471 10.1074/jbc.M109.082958 20106971 PMC2856253

[B16] Chappell J. D. Prota A. E. Dermody T. S. Stehle T. (2002). Crystal structure of reovirus attachment protein sigma1 reveals evolutionary relationship to adenovirus fiber. *EMBO J.* 21 1–11 10.1093/emboj/21.1.1 11782420 PMC125343

[B17] Collins B. Bebeacua C. Mahony J. Blangy S. Douillard F. P. Veesler D. (2013). Structure and functional analysis of the host recognition device of lactococcal phage Tuc2009. *J. Virol.* 87 8429–8440 10.1128/JVI.00907-13 23698314 PMC3719809

[B18] Davidson A. R. Cardarelli L. Pell L. G. Radford D. R. Maxwell K. L. (2012). Long noncontractile tail machines of bacteriophages. *Adv. Exp. Med. Biol.* 726 115–142 10.1007/978-1-4614-0980-9_6 22297512

[B19] De Haard H. J. Bezemer S. Ledeboer A. M. Muller W. H. Boender P. J. Moineau S. (2005). Llama antibodies against a lactococcal protein located at the tip of the phage tail prevent phage infection. *J. Bacteriol.* 187 4531–4541 10.1128/JB.187.13.4531-4541.2005 15968064 PMC1151777

[B20] Desmyter A. Farenc C. Mahony J. Spinelli S. Bebeacua C. Blangy S. (2013). Viral infection modulation and neutralization by camelid nanobodies. *Proc. Natl. Acad. Sci. U.S.A.* 110 E1371–E1379 10.1073/pnas.1301336110 23530214 PMC3625315

[B21] Deveau H. Labrie S. J. Chopin M. C. Moineau S. (2006). Biodiversity and classification of lactococcal phages. *Appl. Environ. Microbiol.* 72 4338–4346 10.1128/AEM.02517-05 16751549 PMC1489595

[B22] Deveau H. Van Calsteren M. R. Moineau S. (2002). Effect of exopolysaccharides on phage-host interactions in *Lactococcus lactis*. *Appl. Environ. Microbiol.* 68 4364–4369 10.1128/AEM.68.9.4364-4369.2002 12200288 PMC124071

[B23] Dube P. Tavares P. Lurz R Van Heel M. (1993). The portal protein of bacteriophage SPP1: a DNA pump with 13-fold symmetry. *EMBO J.* 12 1303–1309 8467790 10.1002/j.1460-2075.1993.tb05775.x PMC413341

[B24] Flayhan A. Vellieux F. M. Lurz R. Maury O. Contreras-Martel C. Girard E. (2014). Crystal structure of pb9, the distal tail protein of bacteriophage T5: a conserved structural motif among all siphophages. *J. Virol.* 88 820–828 10.1128/JVI.02135-13 24155371 PMC3911636

[B25] Flayhan A. Wien F. Paternostre M. Boulanger P. Breyton C. (2012). New insights into pb5, the receptor binding protein of bacteriophage T5, and its interaction with its *Escherichia coli* receptor FhuA. *Biochimie* 94 1982–1989 10.1016/j.biochi.2012.05.021 22659573

[B26] Fokine A. Leiman P. G. Shneider M. M. Ahvazi B. Boeshans K. M. Steven A. C. (2005). Structural and functional similarities between the capsid proteins of bacteriophages T4 and HK97 point to a common ancestry. *Proc. Natl. Acad. Sci. U.S.A.* 102 7163–7168 10.1073/pnas.0502164102 15878991 PMC1129118

[B27] Fraser J. S. Yu Z. Maxwell K. L. Davidson A. R. (2006). Ig-like domains on bacteriophages: a tale of promiscuity and deceit. *J. Mol. Biol.* 359 496–507 10.1016/j.jmb.2006.03.043 16631788

[B28] Hamers-Casterman C. Atarhouch T. Muyldermans S. Robinson G. Hamers C. Songa E. B. (1993). Naturally occurring antibodies devoid of light chains. *Nature* 363 446–448 10.1038/363446a0 8502296

[B29] Jobichen C. Chakraborty S. Li M. Zheng J. Joseph L. Mok Y. K. (2010). Structural basis for the secretion of EvpC: a key type VI secretion system protein from *Edwardsiella tarda*. *PLoS ONE* 5:e12910 10.1371/journal.pone.0012910 PMC2944823 20886112

[B30] Kanamaru S. Leiman P. G. Kostyuchenko V. A. Chipman P. R. Mesyanzhinov V. V. Arisaka F. (2002). Structure of the cell-puncturing device of bacteriophage T4. *Nature* 415 553–557 10.1038/415553a 11823865

[B31] Ledeboer A. M. Bezemer S. De Hiaard J. J. Schaffers I. M. Verrips C. T. Van Vliet C. (2002). Preventing phage lysis of *Lactococcus lactis* in cheese production using a neutralizing heavy-chain antibody fragment from llama. *J. Dairy Sci.* 85 1376–1382 10.3168/jds.S0022-0302(02)74204-5 12146467

[B32] Leiman P. G. Basler M. Ramagopal U. A. Bonanno J. B. Sauder J. M. Pukatzki S. (2009). Type VI secretion apparatus and phage tail-associated protein complexes share a common evolutionary origin. *Proc. Natl. Acad. Sci. U.S.A.* 106 4154–4159 10.1073/pnas.0813360106 19251641 PMC2657435

[B33] Lhuillier S. Gallopin M. Gilquin B. Brasiles S. Lancelot N. Letellier G. (2009). Structure of bacteriophage SPP1 head-to-tail connection reveals mechanism for viral DNA gating. *Proc. Natl. Acad. Sci. U.S.A.* 106 8507–8512 10.1073/pnas.0812407106 19433794 PMC2689013

[B34] Mahony J. Kot W. Murphy J. Ainsworth S. Neve H. Hansen L. H. (2013). Investigation of the relationship between lactococcal host cell wall polysaccharide genotype and 936 phage receptor binding protein phylogeny. *Appl. Environ. Microbiol.* 79 4385–4392 10.1128/AEM.00653-13 23666332 PMC3697520

[B35] Moineau S. Tremblay D. Labrie S. (2002). Phages of lactic acid bacteria: from genomics to industrial applications. *ASM News* 68 388–393

[B36] Mooney D. T. Jann M. Geller B. L. (2006). Subcellular location of phage infection protein (Pip) in *Lactococcus lactis*. *Can. J. Microbiol.* 52 664–672 10.1139/w06-013 16917523

[B37] Muyldermans S. Cambillau C. Wyns L. (2001). Recognition of antigens by single-domain antibody fragments: the superfluous luxury of paired domains. *Trends Biochem. Sci.* 26 230–235 10.1016/S0968-0004(01)01790-X 11295555

[B38] Olson N. H. Gingery M. Eiserling F. A. Baker T. S. (2001). The structure of isometric capsids of bacteriophage T4. *Virology* 279 385–391 10.1006/viro.2000.0735 11162794

[B39] Pedersen M. Ostergaard S. Bresciani J. Vogensen F. K. (2000). Mutational analysis of two structural genes of the temperate lactococcal bacteriophage TP901-1 involved in tail length determination and baseplate assembly. *Virology* 276 315–328 10.1006/viro.2000.0497 11040123

[B40] Pell L. G. Cumby N. Clark T. E. Tuite A. Battaile K. P. Edwards A. M. (2013). A conserved spiral structure for highly diverged phage tail assembly chaperones. *J. Mol. Biol.* 425 2436–2449 10.1016/j.jmb.2013.03.035 23542344

[B41] Pell L. G. Gasmi-Seabrook G. M. Morais M. Neudecker P. Kanelis V. Bona D. (2010). The solution structure of the C-terminal Ig-like domain of the bacteriophage lambda tail tube protein. *J. Mol. Biol.* 403 468–479 10.1016/j.jmb.2010.08.044 20826161

[B42] Pell L. G. Kanelis V. Donaldson L. W. Howell P. L. Davidson A. R. (2009). The phage lambda major tail protein structure reveals a common evolution for long-tailed phages and the type VI bacterial secretion system. *Proc. Natl. Acad. Sci. U.S.A.* 106 4160–4165 10.1073/pnas.0900044106 19251647 PMC2657425

[B43] Plisson C. White H. E. Auzat I. Zafarani A. Sao-Jose C. Lhuillier S. (2007). Structure of bacteriophage SPP1 tail reveals trigger for DNA ejection. *EMBO J.* 26 3720–3728 10.1038/sj.emboj.7601786 17611601 PMC1949002

[B44] Ricagno S. Campanacci V. Blangy S. Spinelli S. Tremblay D. Moineau S. (2006). Crystal structure of the receptor-binding protein head domain from *Lactococcus lactis* phage bIL170. *J. Virol.* 80 9331–9335 10.1128/JVI.01160-06 16940545 PMC1563906

[B45] Ruud V. William E. S. Bruce L. G. (1994). *Lactococcus lactis* ssp. *lactis* C2 bacteriophage sk1 receptor involving rhamnose and glucose moieties in the cell wall. *J. Dairy Sci.* 77 1–6 10.3168/jds.S0022-0302(94)76921-67962868

[B46] Sao-Jose C. Lhuillier S. Lurz R. Melki R. Lepault J. Santos M. A. (2006). The ectodomain of the viral receptor YueB forms a fiber that triggers ejection of bacteriophage SPP1 DNA. *J. Biol. Chem.* 281 11464–11470 10.1074/jbc.M513625200 16481324

[B47] Sassi M. Bebeacua C. Drancourt M. Cambillau C. (2013). The first structure of a mycobacteriophage, the *Mycobacterium abscessus* subsp. *bolletii* phage Araucaria. *J. Virol.* 87 8099–8109 10.1128/JVI.01209-13 PMC3700213 23678183

[B48] Sciara G. Bebeacua C. Bron P. Tremblay D. Ortiz-Lombardia M. Lichiere J. (2010). Structure of lactococcal phage p2 baseplate and its mechanism of activation. *Proc. Natl. Acad. Sci. U.S.A.* 107 6852–6857 10.1073/pnas.1000232107 20351260 PMC2872406

[B49] Sciara G. Blangy S. Siponen M. Mc Grath S. Van Sinderen D. Tegoni M. (2008). A topological model of the baseplate of lactococcal phage Tuc2009. *J. Biol. Chem.* 283 2716–2723 10.1074/jbc.M707533200 18045876

[B50] Shepherd D. A. Veesler D. Lichiere J. Ashcroft A. E. Cambillau C. (2011). Unraveling lactococcal phage baseplate assembly by mass spectrometry. *Mol. Cell. Proteomics* 10, M111.009787. 10.1074/mcp.M111.009787 PMC3186816 21646642

[B51] Siponen M. Sciara G. Villion M. Spinelli S. Lichiere J. Cambillau C. (2009a). Crystal structure of ORF12 from *Lactococcus lactis* phage p2 identifies a tape measure protein chaperone. *J. Bacteriol.* 191 728–734 10.1128/JB.01363-08 19047351 PMC2632072

[B52] Siponen M. Spinelli S. Blangy S. Moineau S. Cambillau C. Campanacci V. (2009b). Crystal structure of a chimeric receptor binding protein constructed from two lactococcal phages. *J. Bacteriol.* 191 3220–3225 10.1128/JB.01637-08 19286807 PMC2687176

[B53] Smith N. L. Taylor E. J. Lindsay A. M. Charnock S. J. Turkenburg J. P. Dodson E. J. (2005). Structure of a group A streptococcal phage-encoded virulence factor reveals a catalytically active triple-stranded beta-helix. *Proc. Natl. Acad. Sci. U.S.A.* 102 17652–17657 10.1073/pnas.0504782102 16314578 PMC1308890

[B54] Soding J. Biegert A. Lupas A. N. (2005). The HHpred interactive server for protein homology detection and structure prediction. *Nucleic Acids Res.* 33 W244–W248 10.1093/nar/gki408 15980461 PMC1160169

[B55] Spinelli S. Campanacci V. Blangy S. Moineau S. Tegoni M. Cambillau C. (2006a). Modular structure of the receptor binding proteins of *Lactococcus lactis* phages. The RBP structure of the temperate phage TP901-1. *J. Biol. Chem.* 281 14256–14262 10.1074/jbc.M600666200 16549427

[B56] Spinelli S. Desmyter A. Verrips C. T. De Haard H. J. Moineau S. Cambillau C. (2006b). Lactococcal bacteriophage p2 receptor-binding protein structure suggests a common ancestor gene with bacterial and mammalian viruses. *Nat. Struct. Mol. Biol.* 13 85–89 10.1038/nsmb1029 16327804

[B57] Stockdale S. R. Mahony J. Courtin P. Chapot-Chartier M. P. Van Pijkeren J. P. Britton R. A. (2013). The lactococcal phages Tuc2009 and TP901-1 incorporate two alternate forms of their tail fiber into their virions for infection specialization. *J. Biol. Chem.* 288 5581–5590 10.1074/jbc.M112.444901 23300085 PMC3581408

[B58] Tam W. Pell L. G. Bona D. Tsai A. Dai X. X. Edwards A. M. (2013). Tail tip proteins related to bacteriophage lambda gpL coordinate an iron-sulfur cluster. *J. Mol. Biol.* 425 2450–2462 10.1016/j.jmb.2013.03.032 23542343 PMC4061613

[B59] Tremblay D. M. Tegoni M. Spinelli S. Campanacci V. Blangy S. Huyghe C. (2006). Receptor-binding protein of *Lactococcus lactis* phages: identification and characterization of the saccharide receptor-binding site. *J. Bacteriol.* 188 2400–2410 10.1128/JB.188.7.2400-2410.2006 16547026 PMC1428394

[B60] Valyasevi R. Sandine W. E. Geller B. L. (1990). The bacteriophage kh receptor of *Lactococcus lactis* subsp. *cremoris* KH is the rhamnose of the extracellular wall polysaccharide. *Appl. Environ. Microbiol.* 56 1882–1889 10.1128/aem.56.6.1882-1889.1990 PMC184526 2116761

[B61] van Raaij M. J. Mitraki A. Lavigne G. Cusack S. (1999). A triple beta-spiral in the adenovirus fibre shaft reveals a new structural motif for a fibrous protein. *Nature* 401 935–938 10.1038/44880 10553913

[B62] van Raaij M. J. Schoehn G. Burda M. R. Miller S. (2001). Crystal structure of a heat and protease-stable part of the bacteriophage T4 short tail fibre. *J. Mol. Biol.* 314 1137–1146 10.1006/jmbi.2000.5204 11743729

[B63] Veesler D. Cambillau C. (2011). A common evolutionary origin for tailed-bacteriophage functional modules and bacterial machineries. *Microbiol. Mol. Biol. Rev.* 75 423–433, first page of table of contents. 10.1128/MMBR.00014-11 21885679 PMC3165541

[B64] Veesler D. Dreier B. Blangy S. Lichiere J. Tremblay D. Moineau S. (2009). Crystal structure and function of a DARPin neutralizing inhibitor of lactococcal phage TP901-1: comparison of DARPin and camelid VHH binding mode. *J. Biol. Chem.* 284 30718–30726 10.1074/jbc.M109.037812 19740746 PMC2781625

[B65] Veesler D. Robin G. Lichiere J. Auzat I. Tavares P. Bron P. (2010). Crystal structure of bacteriophage SPP1 distal tail protein (gp19.1): a baseplate hub paradigm in Gram-positive infecting phages. *J. Biol. Chem.* 285 36666–36673 10.1074/jbc.M110.157529 20843802 PMC2978595

[B66] Veesler D. Spinelli S. Mahony J. Lichiere J. Blangy S. Bricogne G. (2012). Structure of the phage TP901-1 1.8 MDa baseplate suggests an alternative host adhesion mechanism. *Proc. Natl. Acad. Sci. U.S.A.* 109 8954–8958 10.1073/pnas.1200966109 22611190 PMC3384155

[B67] Vinga I. Baptista C. Auzat I. Petipas I. Lurz R. Tavares P. (2012). Role of bacteriophage SPP1 tail spike protein gp21 on host cell receptor binding and trigger of phage DNA ejection. *Mol. Microbiol.* 83 289–303 10.1111/j.1365-2958.2011.07931.x 22171743

[B68] Wikoff W. R. Liljas L. Duda R. L. Tsuruta H. Hendrix R. W. Johnson J. E. (2000). Topologically linked protein rings in the bacteriophage HK97 capsid. *Science* 289 2129–2133 10.1126/science.289.5487.2129 11000116

